# Suicide prevention in France put to the test by COVID-19

**DOI:** 10.1093/eurpub/ckac129.528

**Published:** 2022-10-25

**Authors:** F Jollant

**Affiliations:** Department of Psychiatry, Université Paris-Saclay and Bicêtre Academic, Paris, France

## Abstract

**Introduction:**

Each year, nearly 9,000 people die from suicide in France, and more than 150,000 attempt suicide. In spite of a decrease for the last 30 years, rates of suicide in France remain higher than the European mean. Since 2018, a national suicide prevention strategy of the Ministry of Health has been in place. In 2020, this new strategy has been exposed to the COVID-19 pandemic.

**Methods:**

First, the different actions of the national suicide prevention strategy will be presented, followed by the results of the available data regarding the impact of COVID-19 on suicidal gestures. These data are 1) hospitalizations for self-harm (ICD-10 codes X60 to X-84) from the national health database; 2) calls to poison control centers and 3) visits to the emergency room for a suicide attempt. The latest figures available will be presented.

**Results:**

The analysis of these data compared to 2019 highlights two main periods. Between March and December 2020, a significant decrease in suicide attempts was observed (8.5%), with a rapid drop during the first week of the first confinement in mid-March 2020, in women and men, and in all age groups except old-aged people. Since January 2021, a significant increase in suicide attempts has been observed among teenage girls, including high-lethality acts. Moreover, among the young and the elderly, the figures are now similar to 2019. Only numbers for middle-aged adults continue to decline.

**Conclusions:**

The impact of the COVID-19 pandemic on suicide attempts appears to be variable over time, according to age and gender. The old-aged people and young people, especially adolescent girls seem to have suffered the most from this situation. It is still too early to know whether the new national suicide strategy has had any positive impact. However, the pandemic has highlighted certain weaknesses in the French system, in particular the lack of recent data on mortality by suicide, and the heavy dependence on a fragile mental health medical system.

